# Mechanism of Dietary Variation of Grazing Yaks on Tibetan Plateau: The Role of Seasonal Heterogeneity of Resources

**DOI:** 10.1002/ece3.73132

**Published:** 2026-02-25

**Authors:** Yuning Ru, Yang Liu, A. Allan Degen, Fuyu Shi, Qunying Zhang, Shuai Zheng, Mengyuan Xu, Dehua Wang, Qiang Zhang, Na Guo

**Affiliations:** ^1^ State Key Laboratory of Hulless Barley and Yak Germplasm Resources and Genetic Improvement Tibet Academy of Agricultural and Animal Husbandry Sciences (TAA AS) Lhasa China; ^2^ School of Life Science Shandong University Qingdao Shandong China; ^3^ Desert Animal Adaptations and Husbandry, Wyler Department of Dryland Agriculture, Blaustein Institutes for Desert Research Ben‐Gurion University of the Negev Beer Sheva Israel; ^4^ State Key Laboratory of Grassland and Agro‐Ecosystems, International Centre for Tibetan Plateau Ecosystem Management, College of Ecology Lanzhou University Lanzhou Gansu China

**Keywords:** dietary niche partitioning, foraging behavior, seasonality, Tibetan plateau

## Abstract

Diet composition is a crucial yet understudied, dimension of animal ecology, with seasonal dietary shifts being a key factor in the population dynamics of large herbivores. However, characterizing these variations and their drivers in free‐ranging animals has been challenging due to their high mobility and the diverse plant species in their diet. According to optimal foraging theory, animals select their diet to maximize energy intake, a decision process that involves evaluating the abundance and quality of potential food sources. We determined the seasonal dietary shifts and food network in high‐altitude grazing yaks using DNA metabarcoding targeting the *trn*L region of fecal samples. Seasonal shifts in yak diet composition were structured by resource heterogeneity and influenced by plant community diversity and aboveground biomass. Dietary diversity and richness were greater in winter than summer, while plant community diversity and species richness exhibited opposite trends. This pattern indicated that yaks exhibited the strongest dietary selection during the summer with high resource abundance. Less selection in winter led to more diet dissimilarities, possibly reflecting a compensatory strategy to mitigate energetic deficits by broadening dietary niche breadth and maximizing resource availability under food limitation conditions. The proportion of forbs consumed by yaks was highest in both summer and winter, while the intake of sedges and grasses increased significantly in winter, suggesting that yaks selected high‐protein forbs over grasses or sedges. Our results support the predictions from optimal foraging theory, demonstrating that the energetic basis of dietary selection governs niche width in seasonal environments. Consistent with predictions from optimal foraging theory, our study shows that the energetic drivers of diet selection determine niche breadth in seasonal environments.

## Graphical abstract

Using DNA metabarcoding, we show that Tibetan yaks are highly selective foragers in the resource‐rich summer but switch to a diverse, generalist diet to survive the harsh winter. This flexible foraging strategy is driven by seasonal plant availability, providing strong support for optimal foraging theory and highlighting the critical need to protect winter forage resources for high‐altitude conservation.

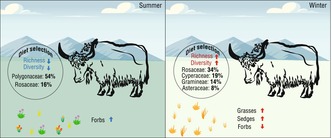

## 1 Introduction

Grazing constitutes the most widespread form of land use globally Maestre's et al. (2022). Grazing herbivores profoundly affect terrestrial ecosystem processes (Staver and Hempson 2020; Trepel et al. 2024) by altering the composition, diversity, and functioning of terrestrial plant communities (Eskelinen et al. 2022). This effect is especially pronounced in alpine grassland, where wild herbivores and livestock can occur at high densities and are highly sensitive to climatic conditions (Zhu et al. 2023). The dietary niche plays a pivotal role in regulating animal density and has profound implications for herbivore coexistence and biodiversity (Kartzinel et al. 2015; Pringle et al. 2019; Pringle and Hutchinson 2020). Despite its importance, understanding the extent of dietary differentiation and mechanisms driving it remains a significant challenge. This is primarily due to the inherent difficulties in accurately determining the diets of herbivores in their natural habitats (Pringle et al. 2023).

Optimal foraging theory (OFT) holds that foraging behavior is driven by the need to maximize the net rate of energy intake, a principle governing decision about both foraging location (marginal value theory; Charnov 1976) and diet composition (basic prey model; Emlen 1966; Stephens and Krebs 1986). A direct consequence of this principle is that foraging selectivity is context‐dependent. Specifically, in environments where high‐quality food is scarce, the theory predicts that animals should adopt a more generalist strategy by consuming readily available, lower‐quality items to reduce the energetic costs associated with searching and thus optimize their overall caloric return (Emlen 1966; Owen‐Smith et al. 2010). Although large herbivores have higher absolute energy requirements, they possess lower mass‐specific metabolic rates than small herbivores and, therefore, could afford to be less selective in their intake (Jarman 1974; Wilson and Kerley 2003).

Shifts in diet selection have been linked to evolutionary differentiation, speciation and environmental gradients (e.g., seasons) (Grant and Grant 2006). Although previous studies examined seasonal dietary variations in small mammals (Sato et al. 2018, 2022), our understanding of these seasonal dynamics in large free‐ranging herbivores remains limited, even though the capacity for dietary flexibility is fundamental to a species' ability to cope with environmental change and influences broader food‐web dynamics (Ducatez et al. 2020; Hutchinson et al. 2021). While various drivers of dietary breadth have been identified for specific taxa (Kartzinel and Pringle 2020; Sato et al. 2018; Westoby 1974), their seasonal dynamics are not well‐characterized. This is particularly true for the yak ( *Bos grunniens* ), an iconic high‐altitude ruminant. Despite the emphasis that OFT models place on diet selection, the seasonal drivers of its foraging choices have been largely overlooked. To address this gap, it is crucial to sample across the full range of seasonal conditions to capture a population's complete dietary spectrum.

Attempts to address patterns of diet selection and niche structure have yielded several key insights. Diet differences among large herbivores arise from the interplay between plant and herbivore traits (Potter et al. 2022), which are the dominant factors in resource partitioning. Even when the available resource base is held constant, the diets of conspecifics can vary significantly with season and geographic location (Parent et al. 2014). For herbivorous animals, dietary selection is primarily shaped by two interacting factors: the seasonal dynamics of food resources and the animal body mass. Seasonal adjustments in diet are a direct response to changes in local forage quality and availability, a flexible strategy that allows herbivores to cope with environmental stressors like drought and can even support population increases (Abraham et al. 2022; Staver and Hempson 2020). Concurrently, body mass exerts a strong influence on foraging strategy; it is positively associated with the quantity of food consumed, the vertical strata exploited for foraging, and the degree of dietary divergence among individuals. Conversely, it shows a negative relationship with the nutritional quality of the diet (Abraham et al. 2022; Codron et al. 2019; Pansu et al. 2022).

The harsh environment of the Tibetan plateau, its complex plant biodiversity, and the high mobility of free‐grazing large herbivores make it challenging to identify and quantify their dietary composition via conventional methods. Previous diet study in free‐ranging herbivores in the region was limited to identifying dominant species using the n‐alkane technique (Ding et al. 2010), which relies on cuticular wax markers, thereby hindering a holistic and nuanced understanding of dietary niche and nutritional intake. Recent advances in molecular approaches enable a finer identification of plant species in the diets of yaks (Guo et al. 2021). However, no previous study has explored the mechanisms underlying seasonal dietary shifts in a free‐ranging high‐altitude herbivore.

Here, we investigated the interaction between environmental factor (seasonal resource variation) and an organismal trait (body mass) on the diet composition and niche breadth of high‐altitude yaks ( *Bos grunniens* ) by analyzing fecal samples using DNA metabarcoding in both summer and winter. Given its high taxonomic resolution and capacity to detect diverse dietary items, DNA metabarcoding proved to be a robust tool for revealing fine‐scale dietary patterns (Alberdi et al. 2019; Ando et al. 2020; Sato 2025), which enabled the identification of seasonal diet shifts and its potential mechanism (Hutchinson et al. 2021; Hoff et al. 2025).

The Tibetan plateau is characterized by large fluctuations in the quantity and quality of available forage. The yak, one of the largest ruminants living at high altitude (Gao et al. 2022), is well adapted to these harsh conditions. With a population of over 18 million, yaks provide the herders with essential resources such as meat, milk, transportation, fuel (from feces), wool, and hides (Qiu et al. 2012). To understand the foraging strategies of this iconic species, we hypothesized that yak diet selection is influenced by the seasonal variation in plant diversity, and we therefore predicted that: (1) yak diets differ in plant species composition between seasons; and (2) these dietary shifts are correlated with seasonal plant diversity.

## 2 Materials and Methods

The studies and all procedures on the yaks were approved by Shandong University: Experimental field management protocol SYDWLL‐2024‐060.

### 2.1 Study Site

**FIGURE 1:**
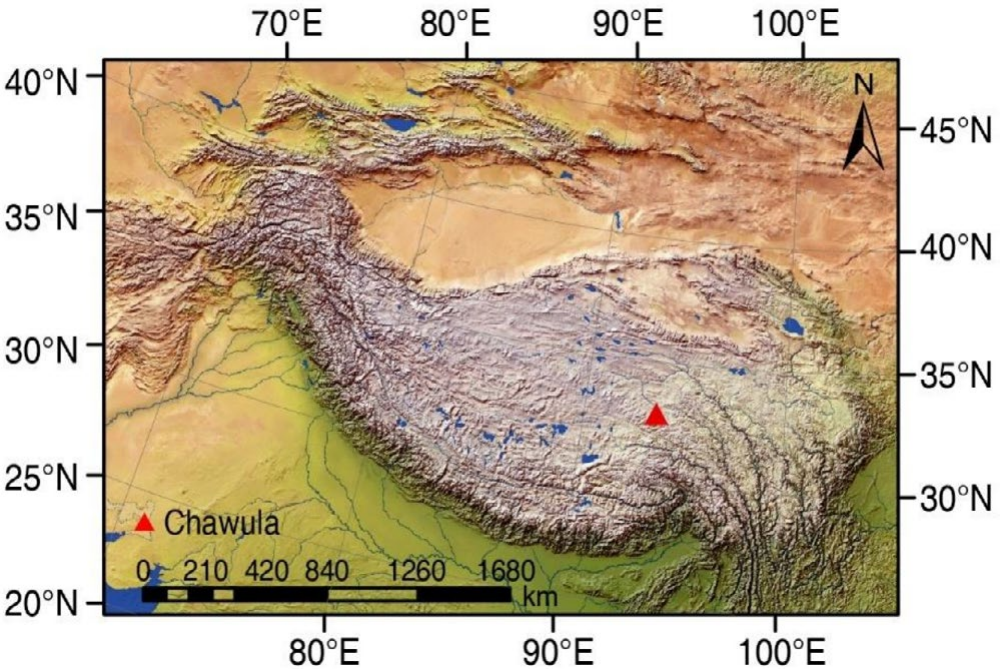
Maps of the study areas and sampling sites. Symbol indicates the localities of sampling sites.

Measurements were made in August (summer) and November (winter), 2023, at the Gala pasture, Nierong county, northern part of the Tibetan plateau (93.59°E, 32.88°N, 4690 m a.s.l., Figure 1). The area is characterized by a long, cold winter, a short, cool summer and a short vegetation growing season. The mean annual air temperature is −2°C and precipitation is 400 mm, with 80% concentrated in the plant growing season from May to September. The vegetation is mainly alpine meadows dominated by *Carex parvula*, *C. kobresia*, *C. capillifolia*, *C. alatauensis*, *Blysmus sinocompressus*, *Ptilagrostis junatovii*, *Potentilla discolor*, *Pedicularis longiflora*, *Leontopodium nanum*, and *Thalictrum alpinum* .

### 2.2 Vegetation Survey and Laboratory Analyses

Above‐ground biomass was harvested in at least six random quadrats (50 cm × 50 cm) within an area of 1.34 ha where yaks grazed, oven‐dried at 65°C to a constant weight, and sorted into the functional groups: forbs, grasses, and sedges.

### 2.3 Yaks and DNA Metabarcoding

In total, 20 grazing yaks, 2.5 years of age, were chosen randomly to measure diet composition in August (*n* = 7) and November (*n* = 13). The seven yaks were weighed in August and November with a walk‐on electronic balance (Weighbridge, Shanghai Jiujin Electronics Apparatus Co. Shanghai, China).

We quantified yak seasonal diet composition using DNA metabarcoding following Pansu et al. (2019). We observed an individual yak until it defecated, collected the fresh feces using nitrile gloves, and placed it in a sterile enzyme‐free centrifuge tube. Samples were transported to the laboratory in liquid nitrogen and subsequently stored at −80°C at Shandong University until DNA extraction. For DNA extraction, the Qiagen QIAamp Fast DNA Stool Mini Kit (Qiagen, Venlo, Netherlands) was employed, with all procedures conducted in a biosafety cabinet. The samples were organized into batches, each containing 49 samples and a negative control to monitor for contamination. Subsequently, a NanoDrop‐2000 UV–Vis Spectrophotometer was used to determine the DNA concentration of each extract (Thermo Scientific, Wilmington, DE, USA).

To identify the plant DNA in our samples, we performed PCR amplification targeting the P6 loop of the chloroplast *trn*L (UAA) intron, which is a standard marker for metabarcoding studies of degraded material and can identify a broad array of plant taxa (Taberlet et al. 2007; Sato 2025). The forward (g): 5′‐GGGCAATCCTGAGCCAA‐3′, reverse (h): 5′‐CCATTGAGTCTCTGCACCTATC‐3 (Taberlet et al. 2007). The primer was modified with Illumina overhang adapters at their 5′ ends. The PCR assays of 10 μL consisted of 0.3 μM of each primer, 0.2 μL KOD FX Neo, 2 μL dNTP, 5 μL KOD FX Neo buffer, and 5 ng of DNA template. In the thermocycling program, there was an initial denaturing at 95°C for 4 min, followed by 30 cycles at 95°C for 30 s, 50°C for 30 s, and 72°C for 60 s, with a 5‐min final extension at 72°C. Following PCR amplification, the products were purified using Agencourt AMPure XP Beads (Beckman Coulter, Indianapolis, IN, USA) to remove primer dimers and non‐specific products. The purified amplicons were amplified using primers containing Illumina indices (i5 and i7) and P5/P7 adapters to index each sample, following the manufacturer's protocol. The final libraries were purified using AMPure XP beads, quantified using the Qubit dsDNA HS Assay Kit (Invitrogen, USA), pooled in equimolar amounts, and sequenced on an Illumina NovaSeq 6000 platform, generating 2 × 150 bp paired‐end reads.

The raw sequencing data were processed using the OBITools v4 pipeline (Coissac 2023). A stringent quality control process was implemented to filter out low‐quality reads. Specifically, sequences were discarded if they exhibited incorrect lengths (≤ 10 bp), contained ambiguous nucleotides (i.e., “N”) or low quality scores (< 30), or were identified as singletons. The high‐quality sequences were clustered into unique sequences using the *obiuniq* command, which groups identical sequences (100% identity), the *obiclean* command was applied to filter out PCR and sequencing errors. Taxonomic identification for each amplicon sequence variants (ASV) was identified primarily by comparison to a local plant DNA reference library from the Tibetan plateau. For sequences where the assignment score with the local library was < 98% (Walker et al. 2023), a secondary comparison was conducted using a global database compiled from the European Molecular Biology Laboratory (EMBL) and National Center for Biotechnology Information (NCBI). The read counts for all retained triplicate PCR replicates of a given sample were averaged. To further reduce noise from potential contaminants or low‐level amplification artifacts, any ASV constituting less than 1% of the total reads within a sample was removed (Guyton et al. 2020). To standardize the sequencing depth for downstream comparisons, all samples were rarefied to a depth of 10,000 reads. Rarefaction analysis indicated that this sequencing depth was sufficient to capture the majority of dietary taxa, as the accumulation curves reached a plateau. From the normalized data, we calculated the relative read abundance (RRA) for each plant taxon within each sample. The RRA is an informative proxy for the proportional biomass of consumed plant taxa and provides a robust picture of diet composition in comparative studies (Kartzinel et al. 2015; Willerslev et al. 2014). While RRA can be affected by several potential sources of error—such as PCR amplification bias, differential digestion rates, or variation in chloroplast density—we contend that any systematic biases should be applied consistently across all our samples. Furthermore, our standardized bioinformatic pipeline and rigorous quality‐control measures are designed to mitigate the impact of any idiosyncratic biases (Deagle et al. 2019). Dietary sequences were categorized into three functional groups: grasses (Gramineae), sedges (Cyperaceae), and forbs (other herbaceous families). Although the *trn*L marker has limited resolution at the species level, it provides robust identification at the family level, allowing for accurate assignment of these broad functional groups.

### 2.4 Statistical Analyses

Wilcoxon test (two sample comparisons) compared functional groups, plant community diversity, above‐ground biomass, dietary richness, and diversity between summer and winter. The effect of season on overall diet composition was statistically evaluated using a permutational multivariate analysis of variance (perMANOVA) based on a Bray–Curtis dissimilarity matrix, running 9999 permutations (“adonis2” in the vegan package in R; Oksanen et al. 2019). Principal Coordinates Analysis (PCoA) was used for visualization of these dietary patterns both within and among individual yaks (Kartzinel et al. 2015; Pansu et al. 2019).

A food network for yaks was constructed to visualize links between yaks in each season and their shared or exclusive diets using the R package bipartite v.2.11. We used Wilcoxon test to compare differences between summer and winter in relative abundance of the five most abundant plant families, as well as the combined relative abundances of the remaining families. We calculated the richness and diversity of dietary taxa within samples based on Hill numbers (^0^ *D* and ^1^ *D*) using the package *vegan* (Oksanen et al. 2019), where ^0^ *D* is species richness (i.e., the number of plant species), ^1^ *D* is species diversity weighted in the diet and equals the exponential of the Shannon index. To identify plant taxa that characterized dietary niche in each season, we performed the indicator species analyses using multipatt function with 999 permutations in package *indicspecies*. This method calculates the association between species and groups (seasons) based on the plant taxa abundance and frequency. Relationships between diet dissimilarity and vegetation diversity/aboveground biomass were tested via linear regression models using the *lm* function in the R packages (https://www.rdocumentation.org/packages/stats/versions/3.5.0). False discovery rate (FDR) correction was applied to all correlation analyses to account for multiple comparisons, with significance defined as an adjusted *p*‐value of < 0.05. All statistical analyses used R 4.1.2 software. Differences were considered significant at *p* < 0.05 unless stated otherwise.

## 3 Results

### 3.1 Seasonal Plant Productivity and Species Composition

**FIGURE 2:**
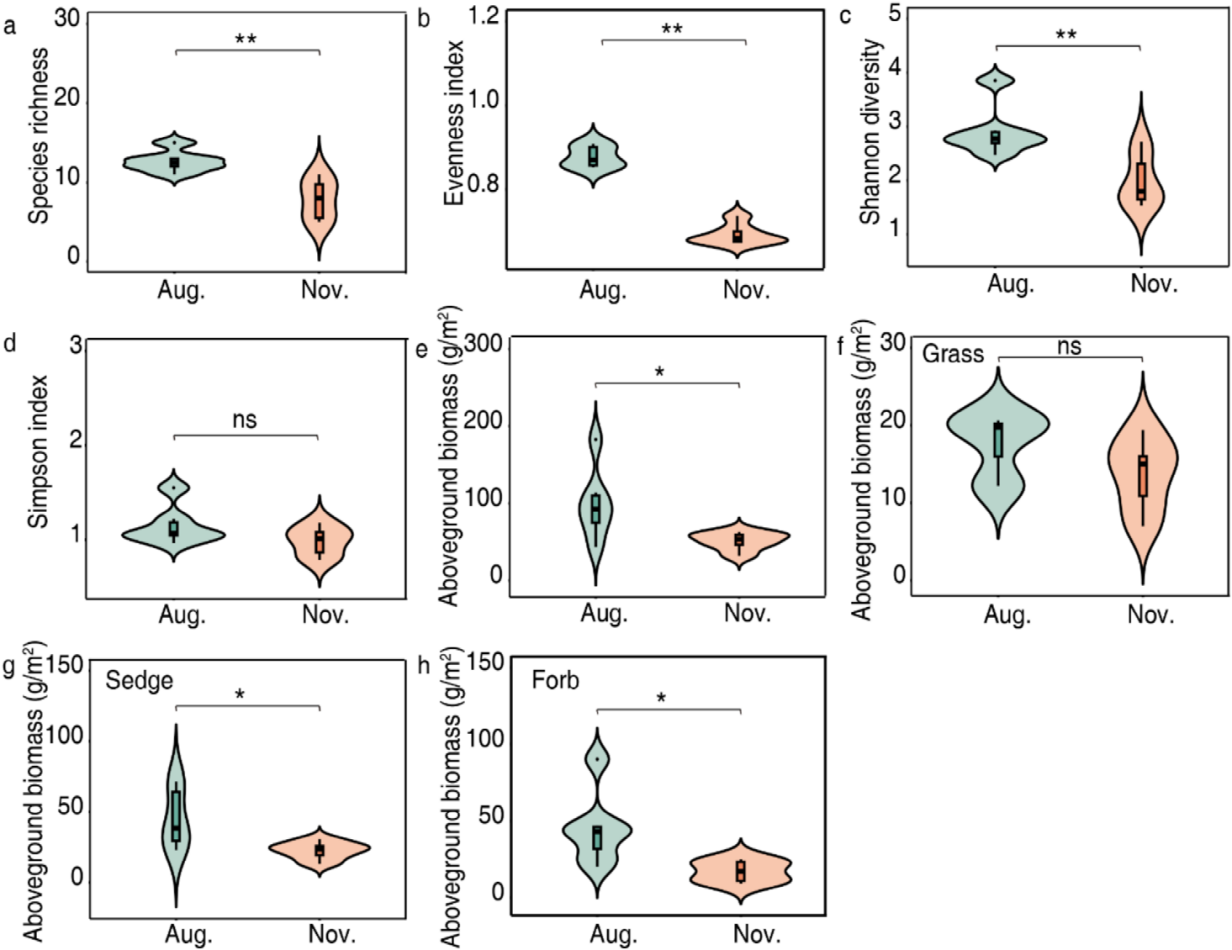
Plant community diversity and biomass of functional groups in summer (August) and winter (November) seasons. (a) species richness, (b) evenness index, (c) Shannon diversity, (d) Simpson diversity, (e) above‐ground biomass, (f) above‐ground biomass of grasses, (g) above‐ground biomass of sedges, (h) above‐ground biomass of forbs in different seasons. ns *p* > 0.05, *p < 0.05, **p < 0.01.

Plant community diversity, including richness, evenness, and Shannon indices, was greater (*p* < 0.01) in summer than in winter (Figure 2a–c). The Simpson index showed no significant difference between seasons (Figure 2d). Aboveground biomass ranged between 32.0 and 182.5 g m^−2^ (average, 75.1 g m^−2^) with greater (*p* < 0.05) biomass in summer than in winter (Figure 2e). Specifically, biomasses of sedges and forbs were greater in summer than in winter (*p* < 0.05, Figure 2g,h), while grasses did not differ between seasons (Figure 2f).

### 3.2 Diet Seasonal Variations

**FIGURE 3:**
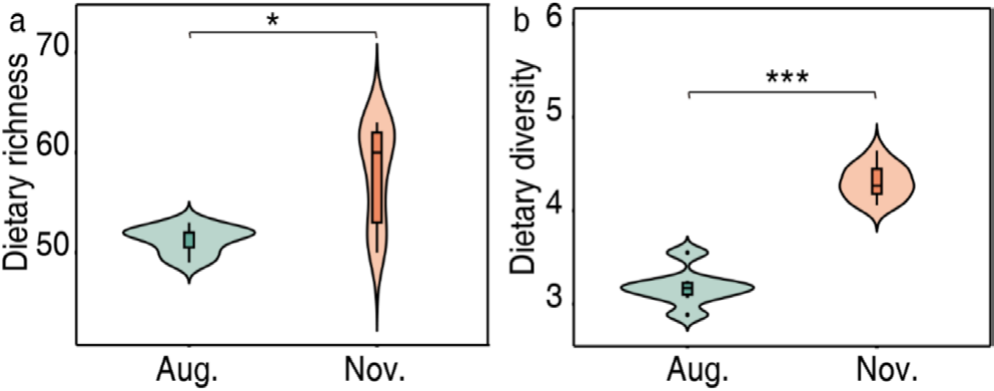
Dietary richness and diversity (Shannon index) between summer (August) and winter (November) seasons (based on the complete set of samples collected in each month). *p < 0.05, ***p < 0.001.

**FIGURE 4:**
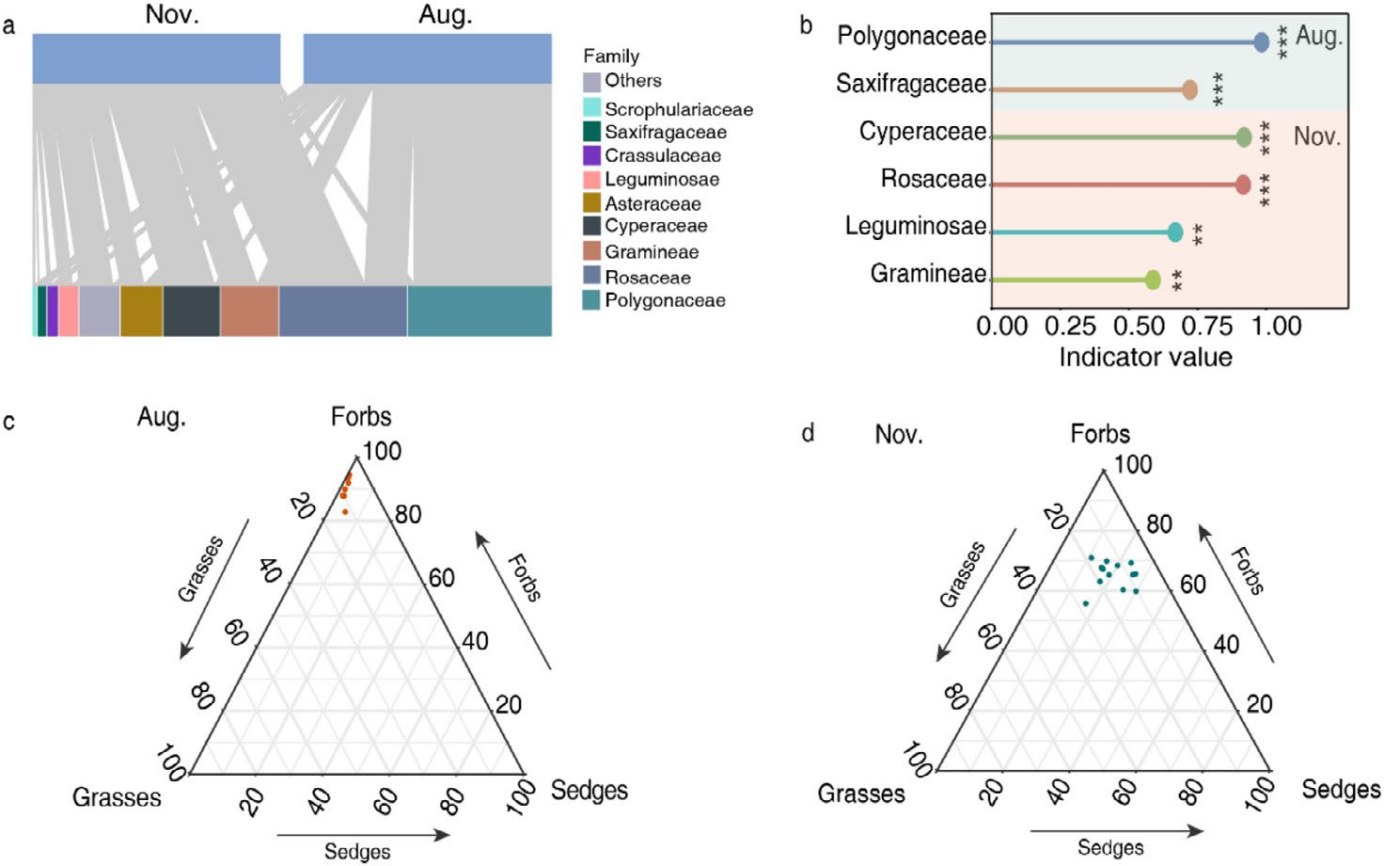
Quantitative food webs and indicator dietary taxa of yak diets on the Tibetan plateau. (a) feeding relationships between yaks (upper boxes) and consumed plant families (lower boxes) in summer (August) and winter (November) seasons. Lines connect yaks in summer and winter (upper boxes) to dietary plant sequences (lower boxes), which are colored by plant family. The widths of the connecting lines represent the relative abundance (RRA) of dietary plant taxa in yak diet in each season. The widths of the lower boxes represent the mean RRA of each dietary plant taxon across all samples from the areas. The widths of the upper bars are season. The mean RRA of the 9 most consumed plant families and the 14 other plant families (RRA > 0.4%). (b) Indicator families across seasons. Colors denote plant families. (c, d) The dietary variation spectrum in plant functional groups between summer and winter. Points display diets of individual yaks. Left axis displays consumption of grasses, bottom axis displays consumption of sedges, and right axis displays consumption of forbs, showing that how taxonomic resolution reveals dimensions of dietary variation. **p < 0.01, ***p < 0.001.

In total, we analyzed plant DNA from 20 fecal samples (7 samples in summer and 13 samples in winter), with seven individuals sampled in both seasons. The average body mass of these seven yaks was 187 (±12) kg in summer and 199 (±13 kg) in winter. Across all samples, we identified 82 unique sequences from 20 plant families. Dietary richness and diversity were greater (*p* < 0.05) in winter than in summer (Figure 3a,b). Yak dietary composition overlapped considerably across seasons (Figure 4), mainly due to Rosaceae, Gramineae, and Asteraceae. There was a clear seasonal shift in their relative read abundance (RRA) (Figure 4a). In summer, the plant families with the highest overall mean RRA were Polygonaceae (forbs, 54%), Rosaceae (forbs, 16%), Gramineae (grasses, 9%), and Asteraceae (forbs, 7%); while in winter, it was Rosaceae (34%), Cyperaceae (sedges, 19%), Gramineae (14%), Asteraceae (8%), and Leguminosae (forbs, 5%). As indicator plant families, Polygonaceae and Saxifragaceae were identified in summer, and Cyperaceae, Rosaceae, Leguminosae, and Gramineae in winter (Figure 4b). At functional group level of the plant taxa, yak diets were dominated by forbs in both seasons, accounting for 90% in summer and 65% in winter. In contrast, the proportion of sedges increased from 2% in summer to 20% in winter, while grasses increased from 9% to 14% (Figure 4c,d).

**FIGURE 5:**
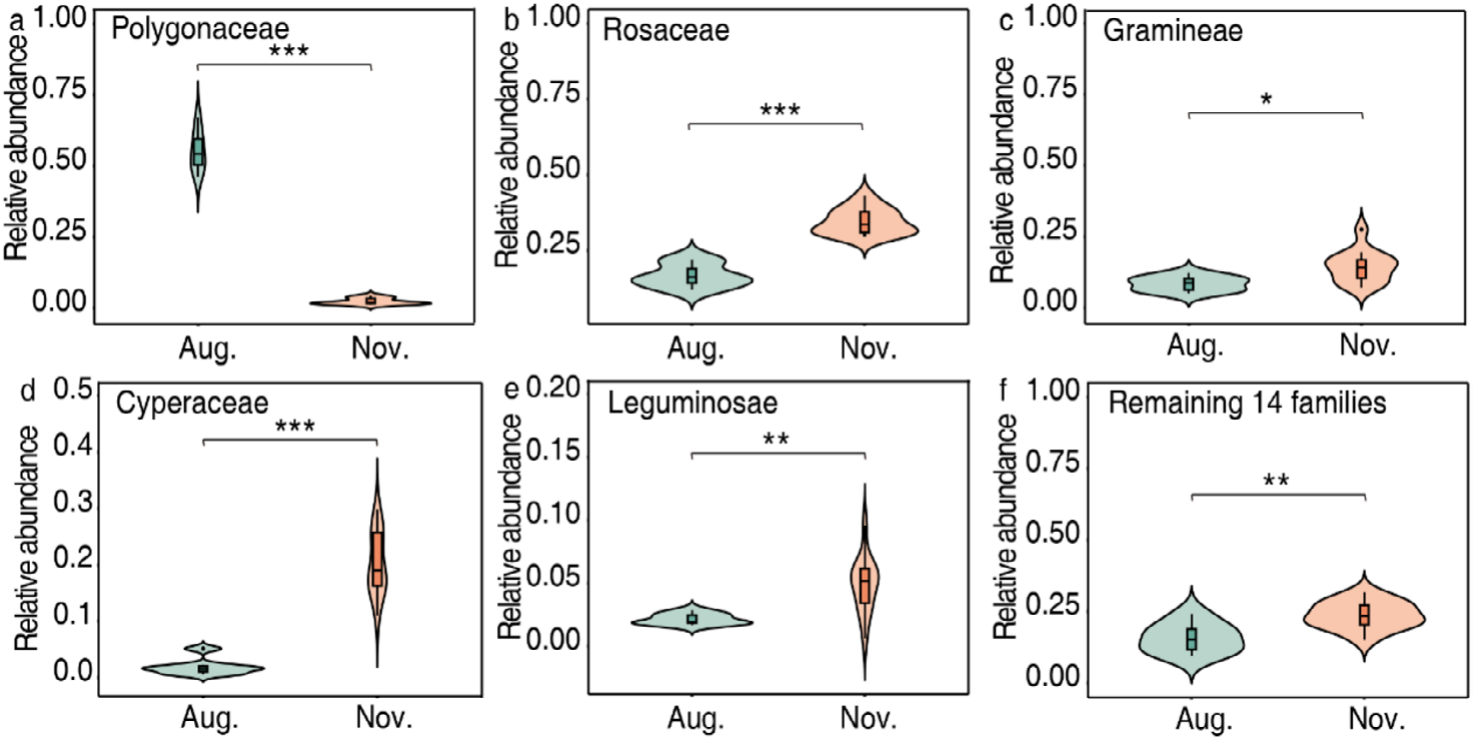
Mean relative abundance of plant families consumed by yaks between seasons. (a) Polygonaceae, (b) Rosaceae, (c) Gramineae, (d) Cyperaceae, (e) Leguminosae, (f) all 14 remaining families. *p < 0.05, **p < 0.01, ***p < 0.001.

To test seasonal dietary selection of the yaks, we compared RRA of the main dietary plant families between summer and winter. Mean RRA of Rosaceae, Gramineae, Cyperaceae, Leguminosae, and the remaining 14 families was greater in winter. Conversely, the consumption of Polygonaceae was less in winter than in summer (Figure 5). The same pattern generally held for the indicator families. These data revealed dietary dynamics of yaks in face of food resource fluctuations between seasons.

### 3.3 Diet Niche Partitioning Across Seasons

**FIGURE 6:**
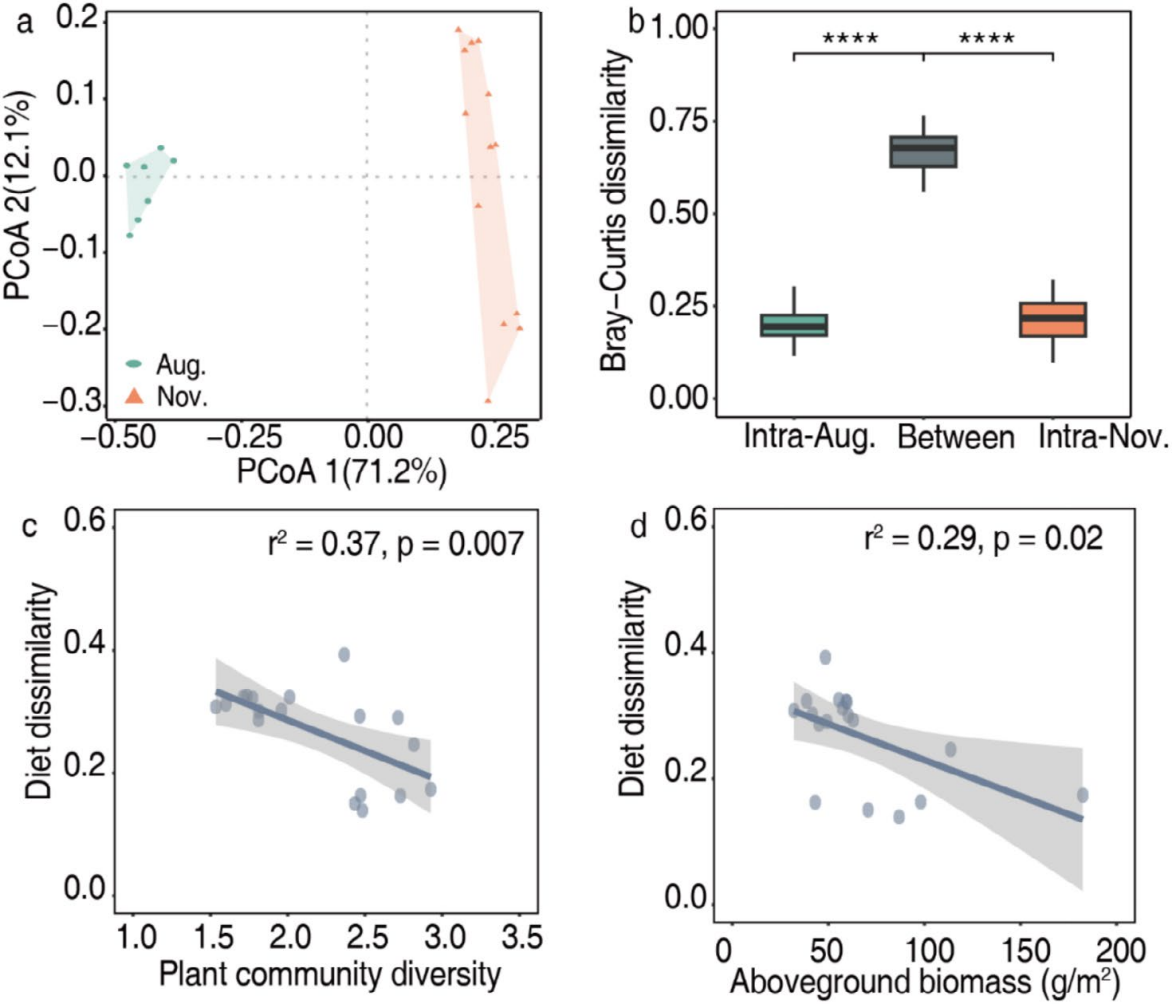
Niche partitioning between summer (August) and winter (November) seasons in yaks. (a) Principal coordinates analysis (PCoA) of molecular dietary inventories of yaks in different seasons using Bray–Curtis dissimilarity. (b) Bray–Curtis dissimilarity between and within seasons. (c) Relationships between yak diet composition and plant community diversity in summer and winter. Blue points illustrate comparisons between yak individuals. The solid line displays a linear regression fit. ****p < 0.0001. (d) Relationships between yak diet composition and aboveground biomass in summer and winter.

To visualize patterns in dietary dissimilarity within and between seasons, we used principal co‐ordinates analysis (PCoA) based on Bray‐Curtis dissimilarities. Yak diet differed between summer and winter (adonis *r* ^2^ = 0.7, *p* = 0.001; Figure 6), as did diet niche (Figure 6a). Accordingly, dietary dissimilarities were lesser within than between seasons (mean dissimilarity = 0.209 vs. 0.669, Figure 6b). Diet composition was influenced by the vegetation diversity index and aboveground biomass, while dietary dissimilarity was correlated negatively to vegetation diversity (*r* ^2^ = 0.37, *p* = 0.007; Figure 6c) and aboveground biomass (*r* ^2^ = 0.29, *p* = 0.02; Figure 6d).

## 4 Discussion

Although patterns of diet variation and specialization have been documented in a number of herbivores (Pringle et al. 2023). Recent studies utilizing DNA metabarcoding have identified specific drivers of dietary shifts, such as interspecific competition (Sato et al. 2018) and seasonal phenology (Sato et al. 2022) in rodents. However, empirical evidence of the mechanisms that drive seasonal differences and their trophic interactions in large high‐altitude free‐ranging herbivores remains limited. By being able to identify individual diet composition with high taxonomic resolution, we demonstrated that yaks differ in dietary diversity and composition between summer and winter due to seasonal heterogeneity in resources available. These findings are consistent with predictions from OFT on energy intake, suggesting a framework for understanding how seasonal resource availability shapes dietary niches and trophic networks.

### 4.1 Seasonal Food Partitioning

Yaks exhibited pronounced seasonal dietary shifts. In summer, their diet was lesser in richness and diversity than in winter, with Polygonaceae, Rosaceae, Cyperaceae, and Gramineae accounting for > 70% of dietary intake. By foraging selectively on abundant and high‐quality plant species, high‐altitude yaks could explain the relationship between seasonal dietary variation and plant community composition. Sedges are the principal native functional group throughout the Tibetan Plateau. Their dominance is driven by their ability to produce high biomass, their wide distribution across the landscape, and their exceptional resistance to grazing and severe climatic events. Although the aboveground biomass of Cyperaceae was greater in summer than in winter, it accounted for only 2% of the yak diet in summer but increased to 20% in winter. Spatio‐seasonal variations in plant nutrient composition and gut microbiome could explain the changes in seasonal sedge consumption for the yaks. Neutral detergent fiber (NDF) content in sedge is greater in winter than summer (Long et al. 1999), and NDF stimulates gut microbiota metabolism and maintains resident bacterial populations. Previous studies demonstrated that *Ruminococcus* spp. improved the degradation of carbohydrates (Flint et al. 2008; Xie et al. 2021), improved fiber digestibility, and is considered an adaptation to poor‐quality diet (Codron et al. 2006).

Grasses are often considered a key dietary component for herbivorous grazers, as was noted across African savannas (Fryxell and Sinclair 1988; Codron et al. 2006). Although yaks are grazers, this did not occur on the alpine grasslands, where grasses comprised a minor component of their diet. Instead, the yak diet was dominated by forbs, comprising 65%–90% of the diet (34%–42% of grassland cover), while grasses comprised 8%–14% of the diet (16%–27% of grassland cover), and sedges comprised 2%–20% of the diet (42%–44% of grassland cover) in both summer and winter. The yaks consumed mainly high‐protein forbs at a proportion substantially greater than the availability. Differences in nutritional quality among plant species might explain the selection for different plant species (Pansu et al. 2019). The forb Polygonaceae is among the top 10 most abundant plant taxa in the study area, and is very rich in protein but also contains phenolic compounds with anti‐nutritive properties. Its heavy consumption during the summer suggests that yaks are capable of ingesting and digesting these plants, and are able to cope with the high contents of phenolic compounds (Menke et al. 1992) or that there are seasonal differences in the phenolic contents. The marked seasonal decrease in the consumption of Polygonaceae in winter was likely due, at least in part, to the loss of leaves caused by snowfall and strong winds. Most heavily selected species, such as *Dasiphora dryadanthoides* and *Potentilla anserina* , have a high protein content and are highly palatable and nutritious, making them preferred by grazers (Long et al. 1999). Although grasses tend to lose their nutritive value rapidly after their growth, nitrogen‐rich forbs such as *Potentilla* spp., when consumed alongside grasses in winter, may help meet the animal's requirements for protein and improve animal performance.

Our study reveals that yaks, typically considered grazers, display significant dietary flexibility to navigate the seasonal shifts in forage quality and availability on the Tibetan Plateau (Fryxell and Sinclair 1988; Abraham et al. 2019). This level of foraging plasticity is analogous to that observed in mixed‐feeding herbivores on the African savanna, whose diets are known to vary dramatically with rainfall‐driven resource cycles (Kartzinel and Pringle 2020; Scanlon et al. 2005; Pansu et al. 2022). Therefore, we propose that a generalist foraging strategy, characterized by a wide dietary niche, is likely an essential adaptation for large herbivores coping with the harsh and variable environment of the Tibetan Plateau.

### 4.2 Drivers of Seasonal Diet Variation

The hypothesis that vegetation diversity and aboveground biomass influenced seasonal dietary selection was supported. As predicted, plant community diversity and richness indices were greater in summer than winter, while dietary diversity and richness followed an opposite trend, that is, greater dietary diversity and richness in winter. This pattern indicates that yaks were more selective (“choosy”) when food resources were abundant, opting for high‐quality, energy‐rich diets in summer. However, when plant community diversity and primary productivity were limited, dietary selection was lesser in winter, but the yaks consumed a more diverse diet. This implies a trade‐off between food quality and energy acquisition, a central tenet of OFT (Emlen 1966; Svanbäck and Bolnick 2005), which is particularly relevant in high‐altitude environments where cold‐season constrains resource availability.

Our study also provides crucial empirical evidence for how resource abundance drives seasonal dietary shifts, a link often highlighted in ecological theories of dietary selection (Stephens and Krebs 1986) and niche differentiation (van Valen 1965) but rarely demonstrated in more seasonal alpine environments with an unproductive cold season (resource‐poor season) followed by a productive warm season (seasons of high resource abundance). Contrary to the simple expectation that greater resource heterogeneity might lead to greater dietary divergence, we found that greater resource abundance is associated with lower dietary dissimilarity among individuals. This pattern emerges because the abundance and accessibility of preferred forage in the warm seasons enabled yaks to select similar high‐quality diets. Conversely, resource scarcity in winter forced yaks to forage more broadly and idiosyncratically, leading to greater dietary dissimilarity. This strategy may help mitigate energetic deficits in winter, a period when free‐grazing yaks are often constrained by food availability, and could reduce food intake and body mass by 30% (Long and Ma 1996). The present study therefore demonstrates that in ecosystems with extreme seasonality, resource availability is the primary driver of dietary niche expansion and diet differentiation.

Results from the study have multiple implications for herbivore ecology in the face of climate change. The dietary flexibility of yaks is key to their survival. As high‐altitude regions warm, altered patterns of snow cover and plant phenology may disrupt the very seasonal resource pulses that these animals have adapted to exploit (Illius and O'Connor 2000; Veldhuis et al. 2019). Understanding this flexibility is therefore essential for predicting their capacity to cope with future environmental change.

## 5 Conclusions

Yaks consistently consumed more forbs than other functional groups but adopted a more diverse, less selective diet in winter when forage quality and quantity were scarce. These seasonal shifts were influenced by plant community diversity and aboveground biomass, providing empirical support for OFT as a framework for understanding how herbivores navigate energetic trade‐offs. Our results highlight the intense selective pressures that extreme seasonality imposes on high‐altitude herbivores, compelling them to adopt a flexible, generalist foraging strategy to survive resource bottlenecks. Therefore, effective conservation and rangeland management on the Tibetan Plateau must account for the seasonal dietary requirements of yaks, particularly by protecting key winter forage resources.

## Author Contributions

**Yuning Ru:** visualization (equal). **Yang Liu:** investigation (equal). **A. Allan Degen:** writing – review and editing (equal). **Fuyu Shi:** visualization (equal). **Qunying Zhang:** investigation (equal). **Shuai Zheng:** visualization (equal). **Mengyuan Xu:** investigation (equal). **Dehua Wang:** writing – review and editing (equal). **Qiang Zhang:** investigation (equal). **Na Guo:** investigation (equal), visualization (equal), writing – original draft (equal), writing – review and editing (equal).

## Funding

This work was supported by the National Natural Science Foundation of China, 32471581.

## Conflicts of Interest

The authors declare no conflicts of interest.

## Data Availability

Data sets utilized for this research are available from Sequence Read Archive (SRA) under PRJNA1321545.
